# What are the best methodologies for rapid reviews of the research evidence for evidence-informed decision making in health policy and practice: a rapid review

**DOI:** 10.1186/s12961-016-0155-7

**Published:** 2016-11-25

**Authors:** Michelle M. Haby, Evelina Chapman, Rachel Clark, Jorge Barreto, Ludovic Reveiz, John N. Lavis

**Affiliations:** 1 Department of Chemical and Biological Sciences, Universidad de Sonora, Hermosillo, Sonora Mexico; 2 Centre for Health Policy, Melbourne School of Population and Global Health, The University of Melbourne, Melbourne, Victoria Australia; 3 Pan American Health Organization, Brasilia, DF Brazil; 4 London School of Hygiene and Tropical Medicine, London, United Kingdom; 5 Fundação Oswaldo Cruz, Diretoria de Brasília, Brasilia, Brazil; 6 Knowledge Management, Bioethics and Research, Pan American Health Organization, Washington, DC, United States of America; 7 McMaster Health Forum, Centre for Health Economics and Policy Analysis, Department of Clinical Epidemiology and Biostatistics, and Department of Political Science, McMaster University, Hamilton, Canada; 8 Department of Global Health and Population, Harvard T.H. Chan School of Public Health, Boston, MA United States of America

**Keywords:** Rapid reviews, Knowledge translation, Evidence-informed decision-making, Research uptake, Health policy

## Abstract

**Background:**

Rapid reviews have the potential to overcome a key barrier to the use of research evidence in decision making, namely that of the lack of timely and relevant research. This rapid review of systematic reviews and primary studies sought to answer the question: What are the best methodologies to enable a rapid review of research evidence for evidence-informed decision making in health policy and practice?

**Methods:**

This rapid review utilised systematic review methods and was conducted according to a pre-defined protocol including clear inclusion criteria (PROSPERO registration: CRD42015015998). A comprehensive search strategy was used, including published and grey literature, written in English, French, Portuguese or Spanish, from 2004 onwards. Eleven databases and two websites were searched. Two review authors independently applied the eligibility criteria. Data extraction was done by one reviewer and checked by a second. The methodological quality of included studies was assessed independently by two reviewers. A narrative summary of the results is presented.

**Results:**

Five systematic reviews and one randomised controlled trial (RCT) that investigated methodologies for rapid reviews met the inclusion criteria. None of the systematic reviews were of sufficient quality to allow firm conclusions to be made. Thus, the findings need to be treated with caution. There is no agreed definition of rapid reviews in the literature and no agreed methodology for conducting rapid reviews. While a wide range of ‘shortcuts’ are used to make rapid reviews faster than a full systematic review, the included studies found little empirical evidence of their impact on the conclusions of either rapid or systematic reviews. There is some evidence from the included RCT (that had a low risk of bias) that rapid reviews may improve clarity and accessibility of research evidence for decision makers.

**Conclusions:**

Greater care needs to be taken in improving the transparency of the methods used in rapid review products. There is no evidence available to suggest that rapid reviews should not be done or that they are misleading in any way. We offer an improved definition of rapid reviews to guide future research as well as clearer guidance for policy and practice.

**Electronic supplementary material:**

The online version of this article (doi:10.1186/s12961-016-0155-7) contains supplementary material, which is available to authorized users.

## Background

In May 2005, the World Health Assembly called on WHO Member States to *“establish or strengthen mechanisms to transfer knowledge in support of evidence-based public health and healthcare delivery systems, and evidence-based health-related policies*” [1]. Knowledge translation has been defined by WHO as: “*the synthesis, exchange, and application of knowledge by relevant stakeholders to accelerate the benefits of global and local innovation in strengthening health systems and improving people’s health*” [2]. Knowledge translation seeks to address the challenges to the use of scientific evidence in order to close the gap between the evidence generated and decisions being made.

To achieve better translation of knowledge from research into policy and practice it is important to be aware of the barriers and facilitators that influence the use of research evidence in health policy and practice decision making [3–8]. The most frequently reported barriers to evidence uptake are poor access to good quality relevant research and lack of timely and relevant research output [7, 9]. The most frequently reported facilitators are collaboration between researchers and policymakers, improved relationships and skills [7], and research that accords with the beliefs, values, interests or practical goals and strategies of decision makers [10].

In relation to access to good quality relevant research, systematic reviews are considered the gold standard and these are used as a basis for products such as practice guidelines, health technology assessments, and evidence briefs for policy [11–14]. However, there is a growing need to provide these evidence products faster and with the needs of the decision-maker in mind, while also maintaining credibility and technical quality. This should help to overcome the barrier of lack of timely and relevant research, thereby facilitating their use in decision making. With this in mind, a range of methods for rapid reviews of the research evidence have been developed and put into practice [15–18]. These often include modifications to systematic review methods to make them faster than a full systematic review. Some examples of modifications that have been made include (1) a more targeted research question/reduced scope; (2) a reduced list of sources searched, including limiting these to specialised sources (e.g. of systematic reviews, economic evaluations); (3) articles searched in the English language only; (4) reduced timeframe of search; (5) exclusion of grey literature; (7) use of search tools that make it easier to find literature; and (7) use of only one reviewer for study selection and/or data extraction. Given the emergence of this approach, it is important to develop a knowledge base regarding the implications of such ‘shortcuts’ on the strength of evidence being delivered to decision makers. At the time of conducting this review, we were not aware of any high quality systematic reviews on rapid reviews and their methods.

It is important to note that a range of terms have been used to describe rapid reviews of the research evidence, including evidence summaries, rapid reviews, rapid syntheses, and brief reviews, with no clear definitions [15, 16, 18–22]. In this paper, we have used the term ‘rapid review’, despite starting with the term ‘rapid evidence synthesis’ in our protocol, as it became clear during the conduct of our review that it is the most widely used term in the literature [23]. We consider a broad range of rapid reviews, including rapid reviews of effectiveness, problem definition, aetiology, diagnostic tests, and reviews of cost and cost-effectiveness.

The rapid review presented in this article is part of a larger project aimed at designing a rapid response program to support evidence-informed decision making in health policy and practice [24]. The expectation is that a rapid response will facilitate the use of research for decision making. We have labelled this study as a rapid review because it was conducted in a limited timeframe and with the needs of health policy decision-makers in mind. It was commissioned by policy decision-makers for their immediate use.

The objective of this rapid review was to use the best available evidence to answer the following research question: What are the best methodologies to enable a rapid review of research evidence for evidence-informed decision making in health policy and practice? Both systematic reviews and primary studies were included. Note that we have deliberately used the term ‘best methodologies’ as it is likely that a variety of methods will be needed depending on the research question, timeframe and needs of the decision maker.

## Methods

This rapid review used systematic review methodology and adheres to the Preferred Reporting Items for Systematic Reviews and Meta-Analysis statement [25]. A systematic review protocol was written and registered prior to undertaking the searches [26]. Deviations from the protocol are listed in Additional file 1.

### Inclusion criteria for studies

Studies were selected based on the inclusion criteria stated below.

### Types of studies

Both systematic reviews and primary studies were sought. For inclusion, priority was given to systematic reviews and to primary studies that used one of the following designs: (1) individual or cluster randomised controlled trials (RCTs) and quasi-randomised controlled trials; (2) controlled before and after studies where participants are allocated to control and intervention groups using non-randomised methods; (3) interrupted time series with before and after measurements (and preferably with at least three measures); and (4) cost-effectiveness/cost-utility/cost-benefit. Other types of studies were also identified for consideration for inclusion in case no systematic reviews and few primary studies with strong study designs (as indicated above in 1–4) could be found. They were initially selected provided that they described some type of evaluation of methodologies for rapid reviews.

### Types of participants

Apart from needing to be within the field of health policy and practice, the types of participants were not restricted, and the level of analysis could be at the level of the individual, organisation, system or geographical area. During the study selection process, we made a decision to also include ‘products’, i.e. papers that include rapid reviews as the unit of inclusion rather than people.

### Types of articles/interventions

Studies that evaluated methodologies or approaches to rapid reviews for health policy and/or practice, including systematic reviews, practice guidelines, technology assessments, and evidence briefs for policy, were included.

### Types of comparisons

Suitable comparisons (where relevant to the article type) included no intervention, another intervention, or current practice.

### Types of outcome measures

Relevant outcome measures included time to complete; resources required to complete (e.g. cost, personnel); measures of synthesis quality; measures of efficiency of methods (measures that combine aspects of quality with time to complete, e.g. limiting data extraction to key characteristics and results that may reduce the time needed to complete without impacting on review quality); satisfaction with methods and products; and implementation. During the study selection process the authors agreed to include two additional outcomes that were not in the published protocol but important for the review, namely comparison of findings between the different synthesis methods (e.g. rapid vs. systematic review) and cost-effectiveness.

Publications in English, French, Portuguese or Spanish, from any country and published from 2004 onwards were included. The year 2004 was chosen as this is the year of the Mexico Ministerial Summit on Health Research, where the know-do gap was first given serious attention by health ministers [27]. Both grey and peer-reviewed literature was sought and included.

### Search methods for identification of studies

A comprehensive search of eleven databases and two websites was conducted. The databases searched were CINAHL, the Cochrane Library (including Cochrane Reviews, the Database of Abstracts of Reviews of Effectiveness, the Health Technology Assessment database, NHS Economic Evaluation Database, and the database of Methods Studies), EconLit, EMBASE, Health Systems Evidence, LILACS and Medline. The websites searched were Google and Google Scholar.

### Grey literature and manual search

Some of the selected databases index a combination of published and unpublished studies (for example, doctoral dissertations, conference abstracts and unpublished reports); therefore, unpublished studies were partially captured through the electronic search process. In addition, Google and Google Scholar were searched. The authors’ own databases of knowledge translation literature were also searched by hand for relevant studies. The reference list of each included study was searched. Contact was made with nine key authors and experts in the area for further studies, of whom five responded.

### Search strategy

Searches were conducted between 15th January and 3rd February 2015 and supplementary searches (reference lists, contact with authors) were conducted in May 2015. Databases were searched using the keywords: “rapid literature review*” OR “rapid systematic review*” or “rapid scoping review*” OR “rapid review*” OR “rapid approach*” OR “rapid synthesis” OR “rapid syntheses” OR “rapid evidence assess*” OR “evidence summar*” OR “realist review*” OR “realist synthesis” OR “realist syntheses” OR “realist evaluation” OR “meta-method*” OR “meta method*” OR “realist approach*” OR “meta-evaluation*” OR “meta evaluation*”. Keywords were searched for in title and abstract, except where otherwise stated in Additional file 2. Results were downloaded into the EndNote reference management program (version X7) and duplicates removed. The Internet search utilised the search terms: “rapid review”; “rapid systematic review”; “realist review”; “rapid synthesis”; and “rapid evidence”.

### Screening and selection of studies

Searches were conducted and screened according to the selection criteria by one review author (MH). The full text of any potentially relevant papers was retrieved for closer examination. This reviewer erred on the side of inclusion where there was any doubt about its inclusion to ensure no potentially relevant papers were missed. The inclusion criteria were then applied against the full text version of the papers (where available) independently by two reviewers (MH and RC). For studies in Portuguese and Spanish, other authors (EC, LR or JB) played the role of second reviewer. Disagreements regarding eligibility of studies were resolved by discussion and consensus. Where the two reviewers were still uncertain about inclusion, the other reviewers (EC, LR, JB, JL) were asked to provide input to reach consensus. All studies which initially appeared to meet the inclusion criteria, but on inspection of the full text paper did not, were detailed in a table ‘Characteristics of excluded systematic reviews,’ together with reasons for their exclusion.

Application of the inclusion criteria by the two reviewers was performed as follows. First, all studies that met the inclusion criteria for participants, interventions and outcomes were selected, providing that they described some type of evaluation of methodologies for rapid evidence synthesis. At this stage, the study type was assessed and categorised by the two reviewers as being a (1) systematic review; (2) primary study with a strong study design, i.e. of one of the four types identified above; or (3) ‘other’ study design (that provided some type of evaluation of methodologies for rapid evidence synthesis). The reason for this was to enable the reviewers to make a decision as to which study designs should be included (based on available evidence, it was not known if sufficient evidence would be found if only systematic reviews and primary studies with strong study designs were included from the outset) and because of interest from the funders in other study types. Following discussion between all co-authors it was decided that it was likely that sufficient evidence could be provided from the first two categories of study type. Thus, the third group was excluded from data extraction but are listed in Additional file 3.

### Data extraction

Information extracted from studies and reviewed included objectives, target population, method/s tested, outcomes reported, country of study/studies and results. For systematic reviews we also extracted the date of last search, the included study designs and the number of studies. For primary studies, we also extracted the year of study, the study design and the population size. Data extraction was performed by one reviewer (MH) and checked by a second reviewer (RC). Disagreements were resolved through discussion and consensus.

### Assessment of methodological quality

The methodological quality of included studies was assessed independently by two reviewers using AMSTAR: A MeaSurement Tool to Assess Reviews [28] for systematic reviews and the Cochrane Risk of Bias Tool for RCTs [29]. Disagreements in scoring were resolved by discussion and consensus. For this review, systematic reviews that achieved AMSTAR scores of 8 to 11 were considered high quality; scores of 4 to 7 medium quality; and scores of 0 to 3 low quality. These cut-offs are commonly used in Cochrane Collaboration overviews. The study quality assessment was used to interpret their results when synthesised in this review and in the formulation of conclusions.

### Data analysis

Findings from the included publications were synthesised using tables and a narrative summary. Meta-analysis was not possible because the included studies were heterogeneous in terms of the populations, methods and outcomes tested.

## Results

### Search results

**Fig. 1:**
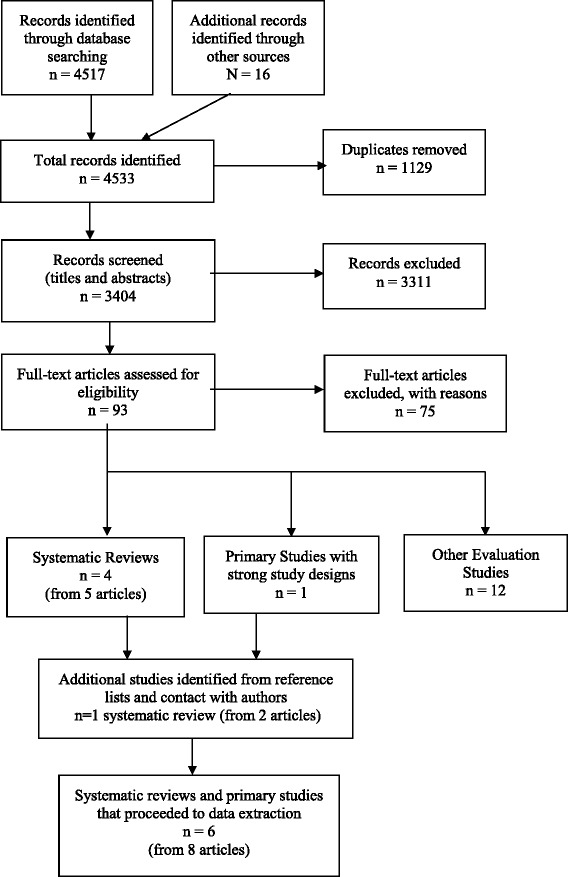
Study selection flow chart – Methods for rapid reviews

Five systematic reviews (from seven articles) [18, 19, 21, 30–33] and one primary study with a strong study design – a RCT [34] – met the inclusion criteria for the review. The selection process for studies and the numbers at each stage are shown in Fig. 1. The reasons for exclusion of the 75 papers at full text stage are shown in Additional file 3. The 12 evaluation studies excluded from data extraction due to weak study designs are also listed at the end of Additional file 3.

**Table 1:** Characteristics of the included systematic reviews. Reviews are ordered chronologically, from most to least recent, and alphabetically within years

Systematic review	Target population	Method/s tested	Included study designs and number	Outcomes reported	AMSTAR score
Featherstone et al., 2015 [31]; Hartling et al., 2015 [32]	Healthcare decision makers	RR – not clearly defined	53 articles: 8 background articles; 3 studies with empiric data; 12 reviews of RR types; 30 articles on RR methods	Type of product; Methods used^a^; Comparison of RRs and SRs	4
Harker & Kleijnen, 2012 [21]	Those making HTA assessments in healthcare	RRs of HTAs	46 full RRs; 3 summaries of RRs	Methods used; Time to complete	2
Abrami et al., 2010 [19]	Policy-makers and practitioners	RRs – defined as a review completed in a timely fashion (i.e. within 6 months) or defined by the authors as such	42 RRs	Methods used	2
Ganann et al., 2010 [18]	Health system planners and policymakers	RRs – undefined	25 RRs; 45 methods articles	Nomenclature; Methods used; Comparison of RRs and SRs; Implications of methods used	2
Cameron et al., 2007 [30]; Watt et al., 2008 [33]	HTA agencies and users	RR, defined as a HTA report or SR that has taken between 1 and 6 months to produce, which contains the elements of a comprehensive literature search	12 studies: 1 guideline (abstract); 3 program evaluations; 2 comparative studies; 2 methods studies; 3 commentaries; 1 survey	RR initiation and rationale; Methods used; Content; Time to complete; Dissemination and impact; Peer review procedures; Quality evaluation of the RR	2

^a^The outcome ‘methods used’ refers to the method used in the included rapid reviews. This outcome is important for determining the quality of the review

*HTA* health technology assessment, *RR* rapid review, *SR* systematic review

### Characteristics of included studies and quality assessment

Characteristics of the included systematic reviews are summarised in Table 1, with full details provided in Additional file 4. All rapid reviews were targeted at healthcare decision makers and/or agencies conducting rapid reviews (including rapid health technology assessments). Only two of the systematic reviews offered a definition of “rapid review” to guide their reviews [19, 30, 33]. Three of the systematic reviews obtained samples of rapid review products – though not necessarily randomly – and examined aspects of the methods used in their production [18, 19, 21]. Three of the systematic reviews reviewed articles on rapid review methods [18, 30, 32]. Two of these also included a comparison of findings from rapid reviews and systematic reviews conducted for the same topic [18, 32].

None of the systematic reviews that were identified examined the outcomes of resources required to complete, synthesis quality, efficiency of methods, satisfaction with methods and products, implementation, or cost-effectiveness. However, while not explicitly assessing synthesis/review quality, all of the reviews did report the methods used to conduct the rapid reviews. We have reported these details as they give an indication of the quality of the review. Therefore, the outcomes reported in the included systematic reviews and recorded in Table 1 and Additional file 4 do not align perfectly with those proposed in our inclusion criteria. In addition, we have included some information that was not pre-defined but for which we extracted information because it provided important contextual information, e.g. type of product, definition, rapid review initiation and rationale, nomenclature, and content. The reporting of the results was also further complicated by the use of a narrative, rather than a quantitative, synthesis of the results in the included studies.

It is not possible to say how many unique studies are included in these systematic reviews because only one review actually included a list of included studies [30] and one a characteristics of included studies table (but not in a form that was easy to use) [21]. However, it is clear that there is likely to be significant overlap in studies between reviews. For example, the most recent systematic review by Hartling et al. [31, 32] also included the four previous systematic reviews included in this rapid review [18, 19, 21, 30, 33].

The RCT was targeted at healthcare professionals involved in clinical guideline development [34]. It aimed to assess the effectiveness of different evidence summary formats for use in clinical guideline development. Three different packs were tested – pack A: a systematic review alone; pack B: a systematic review with summary-of-findings tables included; and pack C: an evidence synthesis and systematic review. Pack C is described by the authors of the study as: “*a locally prepared, short, contextually framed, narrative report in which the results of the systematic review (and other evidence where relevant) were described and locally relevant factors that could influence the implementation of evidence-based guideline recommendations (e.g. resource capacity) were highlighted*” [34]. We interpreted pack C as being a ‘rapid review’ for the purposes of this review as the authors state that it is based on a comprehensive search and critical appraisal of the best currently available literature, which included a Cochrane review, an overview of systematic reviews and RCTs, and additional RCTs but was likely to have been done in a short timeframe. It was also conducted to help improve decision-making. The primary outcome measured was the proportion of correct responses to key clinical questions, whilst the secondary outcome was a composite score comprised of clarity of presentation and ease of locating the quality of evidence [34]. This study was not included in any previous systematic reviews.

Four of the systematic reviews obtained AMSTAR scores of 2 (low quality) and one a score of 4 (medium quality). No high quality systematic reviews were found. Thus, the findings of the systematic reviews should be taken as indicative only and no firm conclusions can be made. The RCT was classified as low risk of bias on the Cochrane Risk of Bias tool. The quality assessments can be found in Additional file 5.

### Findings

#### Definition of a ‘rapid review’

The five systematic reviews are consistent in stating that there is no agreed definition of rapid reviews and no agreed methodology for conducting rapid reviews [18, 19, 21, 30–33]. According to the authors of one review: “*the term ‘rapid review’ does not appear to have one single definition but is framed in the literature as utilizing various stipulated time frames between 1 and 6 months*” [21, p. 398]. The definitions offered to guide the reviews by Abrami et al. [19] and Cameron et al. [30] both use a timeframe of up to 6 months (Table 1). Cameron et al. [30] also include in their definition the requirement that the review contains the elements of a comprehensive search – though they do not offer criteria to assess this.

Abrami et al. [19] use the term ‘brief review’ rather than ‘rapid review’ to emphasise that both timeframe and scope may be affected. They write that “*a brief review is an examination of empirical evidence that is limited in its timeframe (e.g. six months or less to complete) and/or its scope, where scope may include:*

- *the breadth of the question being explored (e.g. a review of one-to-one laptop programs versus a review of technology integration in schools);*
- *the timeliness of the evidence included (e.g. the last several years of research versus no time limits);*
- *the geographic boundaries of the evidence (e.g. inclusion of regional or national studies only versus international evidence);*
- *the depth and detail of analyses (e.g. reporting only overall findings versus also exploring variability among the findings); or*
- *otherwise more restrictive study inclusion criteria than might be seen in a comprehensive review*.” [19, p. 372].

All other included systematic reviews used the term ‘rapid review’ or ‘rapid health technology assessment’ to describe rapid reviews.

### Methods used based on examples of rapid reviews

While the word ‘rapid’ indicates that it will be carried out quickly, there is no consistency in published rapid reviews as to how it is made rapid and which part, or parts, of the review are carried out at a faster pace than a full systematic review [18, 19, 21]. A further complexity is the reporting of methods used in the rapid review, with about 43% of the rapid reviews examined by Abrami et al. [19] not describing their methodology comprehensively. Three examples of ‘shortcuts’ taken are (1) not using two reviewers for study selection and/or data extraction; (2) not conducting a quality assessment of included studies; and (3) not searching for grey literature [18, 19, 21]. However, it is important to note that the rapid reviews examined in these three systematic reviews were not necessarily selected randomly and, thus, it is not possible to accurately quantify the proportion of rapid reviews taking various ‘shortcuts’ and which ‘shortcuts’ are the most common. The time taken for the reviews examined varied from several days to one year [19]; 3 weeks to 6 months [18]; and 7–12 (mean 10.42, SD 7.1) months [21].

### Methods used based on studies of rapid review methods

Methodological approaches or ‘shortcuts’ used in rapid reviews to make them faster than a full systematic review include [18, 19, 32] limiting the number of questions; limiting the scope of questions; searching fewer databases; limited use of grey literature; restricting the types of studies included (e.g. English only, most recent 5 years); relying on existing systematic reviews; eliminating or limiting hand searching of reference lists and relevant journals; narrow time frame for article retrieval; using non-iterative search strategy; eliminating consultation with experts; limiting full-text review; limiting dual review for study selection, data extraction and/or quality assessment; limiting data extraction; limiting risk of bias assessment or grading; minimal evidence synthesis; providing minimal conclusions or recommendations; and limiting external peer review. Harker et al. [21] found that, with increasing timeframes, fewer of the ‘shortcuts’ were used and that, with longer timeframes, it was more likely that risk of bias assessment, evidence grading and external peer review would be conducted [21].

None of the included systematic reviews offer firm guidelines for the methodology underpinning rapid reviews. Rather, they report that many articles written about rapid reviews offer only examples and discussion surrounding the complexity of the area [30].

### Supporting evidence for shortcuts

While authors of the included systematic reviews tend to agree that changes to scope or timeframe can introduce biases (e.g. selection bias, publication bias, language of publication bias) they found little empirical evidence to support or refute that claim [18, 19, 21, 30, 32].

The review by Ganann et al. [18] included 45 methodological studies that considered issues such as the impact of limiting the number of databases searched, hand searching of reference lists and relevant journals, omitting grey literature, only including studies published in English, and omitting quality assessment. However, they were unable to provide clear methodological guidelines based on the findings of these studies.

### Comparison of findings – rapid reviews versus systematic reviews

A key question is whether the conclusions of a rapid review are fundamentally different to a full systematic review, i.e. whether they are sufficiently different to change the resulting decision. This is an area where the research is extremely limited. There are few comparisons of full and rapid reviews that are available in the literature to be able to determine the impact of the above methodological changes – only two primary studies were reported in the included systematic reviews [35, 36]. It is important to note that neither of these studies met, on their own, the inclusion criteria for the review in that they did not have a sufficiently strong study design. Both are included in the list of 12 studies excluded from data extraction (Fig. 1 and Additional file 3). Thus, they provide a very low level of evidence.

One of the primary studies compared full and rapid reviews on the topics of drug eluting stents, lung volume reduction surgery, living donor liver transplantation and hip resurfacing [30, 36]. There were no instances in which the essential conclusions of the rapid and full reviews were opposed [32]. The other compared a rapid review with a full systematic review on the use of potato peels for burns [35]. The results and conclusions of the two reports were different. The authors of the rapid review suggest that this is because the systematic review was not of sufficiently good quality – as they missed two important trials in their search [35]. However, the limited detail on the methods used to conduct the systematic review makes this case study of limited value. Further research is needed in this area.

### Impact of rapid syntheses on understanding of decision makers

The included RCT by Opiyo et al. [34] examined the impact of different evidence summary or synthesis formats on knowledge of the evidence, with each participant receiving a pack containing three different summaries; they found no differences between packs in the odds of correct responses to key clinical questions. Pack C (the rapid review) was associated with a higher mean composite score for clarity and accessibility of information about the quality of evidence for critical neonatal outcomes compared to systematic reviews alone (pack A) (adjusted mean difference 0.52, 95% confidence interval, 0.06–0.99). Findings from interviews with 16 panellists indicated that short narrative evidence reports (pack C) were preferred for the improved clarity of information presentation and ease of use. The authors concluded that their “*findings suggest that ‘graded-entry’ evidence summary formats may improve clarity and accessibility of research evidence in clinical guideline development*” [34, p. 1].

## Discussion

This review is the first high quality review (using systematic reviews as the gold standard for literature reviews) published in the literature that provides a comprehensive overview of the state of the rapid review literature. It highlights the lack of definition, lack of defined methods and lack of research evidence showing the implications of methodological choices on the results of both rapid reviews and systematic reviews. It also adds to the literature by offering clearer guidance for policy and practice than has been offered in previous reviews (see Implications for policy and practice).

While five systematic reviews of methods for rapid reviews were found, none of these were of sufficient quality to allow firm conclusions to be made. Thus, the findings need to be treated with caution. There is no agreed definition of rapid reviews in the literature and no agreed methodology for conducting rapid reviews [18, 19, 21, 30–33]. However, the systematic reviews included in this review are consistent in stating that a rapid review is generally conducted in a shorter timeframe and may have a reduced scope. A wide range of ‘shortcuts’ are used to make rapid reviews faster than a full systematic review. While authors of the included systematic reviews tend to agree that changes to scope or timeframe can introduce biases (e.g. selection bias, publication bias, language of publication bias) they found little empirical evidence to support or refute that claim [18, 19, 21, 30, 32]. Further, there are few comparisons available in the literature of full and rapid reviews to be able to determine the impact of these ‘shortcuts’. There is some evidence from a good quality RCT with low risk of bias that rapid reviews may improve clarity and accessibility of research evidence for decision makers [34], which is a unique finding from our review.

A scoping review published after our search found over 20 different names for rapid reviews, with the most frequent term being ‘rapid review’, followed by ‘rapid evidence assessment’ and ‘rapid systematic review’ [23]. An associated international survey of rapid review producers and modified Delphi approach counted 31 different names [37]. With regards to rapid review methods and definitions, the scoping review found 50 unique methods, with 16 methods occurring more than once [23]. For their scoping review and international survey, Tricco et al. utilised the working definition: “*a rapid review is a type of knowledge synthesis in which components of the systematic review process are simplified or omitted to produce information in a short period of time*” [23, 37].

The authors of the most recent systematic review of rapid review methods suggest that: “*the similarity of rapid products lies in their close relationship with the end-user to meet decision making needs in a limited timeframe*” [32, p. vii]. They suggest that this feature drives other differences, including the large range of products often produced by rapid response groups, and the wide variation in methods used [32] – even within the same product type produced by the same group. We suggest that this feature of rapid reviews needs to be part of the definition and considered in future research on rapid reviews, including whether it actually leads to better uptake of research. To aid future research, we propose the following definition: a rapid review is a type of systematic review in which components of the systematic review process are simplified, omitted or made more efficient in order to produce information in a shorter period of time, preferably with minimal impact on quality. Further, they involve a close relationship with the end-user and are conducted with the needs of the decision-maker in mind.

When comparing rapid reviews to systematic reviews, the confounding effects of quality of the methods used must be considered. If rapid syntheses of research are seen as systematic reviews performed faster and if systematic reviews are seen as the gold standard for evidence synthesis, the quality of the review is likely to depend on which ‘shortcuts’ were taken and this can be assessed using available quality measures, e.g. AMSTAR [28]. While Cochrane Collaboration systematic reviews are consistently of a very high quality (achieving 10 or 11 on the AMSTAR scale, based on our own experience) the same cannot be said for all systematic reviews that can be found in the published literature or in databases of systematic reviews – as is demonstrated by this review where AMSTAR scores were quite low (Additional file 5) and a related overview where AMSTAR scores varied between two and ten [24, Additional file one]. This fact has not been acknowledged in previous syntheses of the rapid review literature. It is also possible for rapid reviews to achieve high AMSTAR scores if conducted and reported well. Therefore, it can be easily argued that a high quality rapid review is likely to provide an answer closer to the ‘truth’ than a systematic review of low quality. It is also an argument for using the same tool for assessing the quality of both systematic and rapid reviews.

Authors of the published systematic reviews of rapid reviews suggest that, rather than focusing on developing a formalised methodology, which may not be appropriate, researchers and users should focus on increasing the transparency of the methods used for each review [18, 30, 33]. Indeed, several AMSTAR criteria are highly dependent on the transparency of the write-up rather than the methodology itself. For example, there are many examples of both systematic and rapid review authors not stating that they used a protocol for their review when, in fact, they did use one, leading to a loss of 1 point on the AMSTAR scale. Another example is review authors failing to provide an adequate description of the study selection and data extraction process, thus making it hard for those assessing the quality of the review to determine if this was done in duplicate, which is again a loss of 1 point on the AMSTAR scale.

While it could be argued that none of the included reviews described their review as a systematic review, we believe that it is appropriate to assess their quality using the AMSTAR tool. This the best tool available, to our knowledge, to assess and compare the quality of review methods and considers the major potential sources for bias in reviews of the literature [28, 38]. Further, the five reviews included were clearly not narrative reviews as each described their methods, including sources of studies, search terms and inclusion criteria used.

### Strengths and limitations

A key strength of this rapid review is the use of high quality systematic review methodology, including the consideration of the scientific quality of the included studies in formulating conclusions. A meta-analysis was not possible due to the heterogeneity in terms of intervention types and populations studied in the included systematic reviews. As a result publication bias could not be assessed quantitatively in this review and no clear methods are available for assessing publication bias qualitatively [39]. Shortcuts taken to make this review more rapid, as well as an AMSTAR assessment of the review, are shown in Additional file 6. The AMSTAR assessment is based on the published tool [28] and additional guidance provided on the AMSTAR website (http://amstar.ca/Amstar_Checklist.php).

The current rapid review is evidence that a review can include several shortcuts and be produced in a relatively short amount of time without sacrificing quality, as shown by the high AMSTAR score (Additional file 6). The time taken to complete this review was 7 months from signing of contract (November 2014) to submission of the final report to the funder (June 2015). Alternatively, if publication of the protocol on PROSPERO and the start of literature searching (January 2014) are taken as the starting point, the time taken was 5 months.

Limitations of this review include (1) the low quality of the systematic reviews found, with three of the four included systematic reviews judged as low quality on the AMSTAR criteria and the fourth just making it to medium quality (Additional file 5); (2) the fact that few primary studies were conducted in developing countries, which is an issue for the generalisability of the results; and (3) restricting the search to articles in English, French, Spanish or Portuguese (languages with which the review authors are competent) and to the last 10 years. However, this was done to expedite the review process and is unlikely to have resulted in the loss of important evidence.

### Implications for policy and practice

Users of rapid reviews should request an AMSTAR rating and a clear indication of the shortcuts taken to make the review process faster. Producers of rapid reviews should give greater consideration to the ‘write-up’ or presentation of their reviews to make their review methods more transparent and to enable a fair quality assessment. This could be facilitated by including the appropriate elements in templates and/or guidelines. If a shorter report is required, the necessary detail could be placed in appendices.

When deciding what methods and/or process to use for their rapid reviews, producers of rapid reviews should give priority to shortcuts that are unlikely to impact on the quality or risk of bias of the review. Examples include limiting the scope of the review [19], limiting data extraction to key characteristics and results [32], and restricting the study types included in the review [32]. When planning the rapid review, the review producer should explain to the user the implications of any shortcuts taken to make the review faster, if any.

Producers of rapid reviews should consider maintaining a larger highly skilled and experienced staff, who can be mobilised quickly, and understands the type of products that might meet the needs of the decision maker [19, 32]. Consideration should also be given to making the process more efficient [19]. These measures can aid timelines without compromising quality.

### Implications for research

The impact on the results of rapid reviews (and systematic reviews) of any ‘shortcuts’ used requires further research, including which ‘shortcuts’ have the greatest impact on the review findings. Tricco et al. [23, 37] suggest that this could be examined through a prospective study that compares the results of rapid reviews to those obtained through systematic reviews on the same topic. However, to do this, it will be important to consider quality as a confounding factor and ensure random selection and blinding of the rapid review producers. If random selection and blinding cannot be guaranteed, we suggest that retrospective comparisons may be more appropriate. Another, related approach, would be to compare findings of reviews (be they systematic or rapid) for each type of shortcut, controlling for methodological quality. Other issues, such as the breadth of the inclusion criteria used and number of studies included would also need to be considered as possible confounding factors.

The development of reporting guidelines for rapid reviews, as are available for full systematic reviews, would also help [18, 25]. These should be heavily based on systematic review guidelines but also consider characteristics specific to rapid reviews such as the relationship with the review user.

Finally, future studies and reviews should also address the outcomes of review quality, satisfaction with methods and products, implementation and cost-effectiveness as these outcomes were not measured in any of the included studies or reviews. Effectiveness of rapid reviews in increasing the use of research evidence in policy decision-making is also an important area for further research.

## Conclusions

Care needs to be taken in interpreting the results of this rapid review on the best methodologies for rapid review given the limited state of the literature. There is a wide range of methods currently used for rapid reviews and wide range of products available. However, greater care needs to be taken in improving the transparency of the methods used in rapid review products to enable better analysis of the implications of methodological ‘shortcuts’ taken for both rapid reviews and systematic reviews. This requires the input of policymakers and practitioners, as well as researchers. There is no evidence available to suggest that rapid reviews should not be done or that they are misleading in any way.

## Additional files

Additional file 1: Changes to the protocol. (DOCX 28 kb)
Additional file 2: Search terms. (DOCX 31 kb)
Additional file 3: Characteristics of excluded studies and reference details. (DOCX 40 kb)
Additional file 4: Characteristics of included studies. (DOCX 47 kb)
Additional file 5: Quality assessment of included studies. (DOCX 29 kb)
Additional file 6: Shortcuts and quality assessment of this review. (DOCX 30 kb)

## Abbreviations

AMSTAR: A MeaSurement Tool to Assess Reviews
PAHO: Pan American Health Organization
PRISMA: Preferred reporting items for systematic reviews and meta-analysis
RCT: Randomised controlled trial
