# Supplementary material for: What are the best methodologies for rapid reviews of the research evidence for evidence-informed decision making in health policy and practice: a rapid review
Source: Health Res Policy Syst. 2016 Nov 25;14:83. doi: 10.1186/s12961-016-0155-7 (PMC5123411; doi:10.1186/s12961-016-0155-7)
Supplement: Additional file 1: — Changes to the protocol. (DOCX 28 kb) [file 12961_2016_155_MOESM1_ESM.docx]

## Additional file 1. Changes to the protocol

### Selection criteria:

- Clarifying that for ‘types of participants’ they needed to be within the field of health policy and practice.
- The search years were not limited to December 2014 and articles published in 2015 were included.
- During the study selection process the authors agreed to include two additional outcomes that were not in the published protocol but important for the review: comparison of findings between the different synthesis methods (e.g. rapid vs systematic review); and cost-effectiveness.

### Search:

- Medline (Ovid) was used instead of PubMed as PubMed did not allow the use of truncation characters in phrases.

### Data extraction:

- Though part of the original protocol, we did not extract data on critical success factors, limitations and research gaps relevant to each included systematic review or primary study. This was due to time and resource limitations.
