# Supplementary material for: What are the best methodologies for rapid reviews of the research evidence for evidence-informed decision making in health policy and practice: a rapid review
Source: Health Res Policy Syst. 2016 Nov 25;14:83. doi: 10.1186/s12961-016-0155-7 (PMC5123411; doi:10.1186/s12961-016-0155-7)
Supplement: Additional file 2: — Search terms. (DOCX 31 kb) [file 12961_2016_155_MOESM2_ESM.docx]

## Additional file 2. Search terms

| Database and search string |
| --- |
| **CINAHL (EBSCOHost)**  TI ( “realist review*” OR “realist synthesis” OR “realist syntheses” OR “realist evaluation” OR “meta-method*” OR “meta method*” OR “realist approach*” OR “meta-evaluation*” OR “meta evaluation*” OR “rapid literature review*” OR “rapid systematic review*” or “rapid scoping review*” OR “rapid review*” or “rapid approach*” or “rapid synthesis” OR “rapid syntheses” OR “rapid evidence assess*” OR “evidence summar*” ) OR AB ( “realist review*” OR “realist synthesis” OR “realist syntheses” OR “realist evaluation” OR “meta-method*” OR “meta method*” OR “realist approach*” OR “meta-evaluation*” OR “meta evaluation*” OR “rapid literature review*” OR “rapid systematic review*” or “rapid scoping review*” OR “rapid review*” or “rapid approach*” or “rapid synthesis” OR “rapid syntheses” OR “rapid evidence assess*” OR “evidence summar*” )  **Limiters** - Published Date: 20040101-20151231 |
| **Cochrane Library**  #1 realist next review:ti,ab,kw or realist next synthes?s:ti,ab,kw or "realist evaluation":ti,ab,kw or meta-method:ti,ab,kw or meta next method:ti,ab,kw  #2 realist next approach*:ti,ab,kw or meta-evaluation*:ti,ab,kw or meta next evaluation*:ti,ab,kw or rapid next literature next review*:ti,ab,kw or rapid next systematic next review*:ti,ab,kw  #3 rapid next scoping next review:ti,ab,kw or rapid next review*:ti,ab,kw or rapid next approach*:ti,ab,kw or rapid next synthes?s:ti,ab,kw or rapid next evidence next assess*:ti,ab,kw  #4 evidence next summar*:ti,ab,kw  #5 #1 or #2 or #3 or #4 |
| **EconLit (EBSCOHost)**  TI ( “realist review*” OR “realist synthesis” OR “realist syntheses” OR “realist evaluation” OR “meta-method*” OR “meta method*” OR “realist approach*” OR “meta-evaluation*” OR “meta evaluation*” OR “rapid literature review*” OR “rapid systematic review*” or “rapid scoping review*” OR “rapid review*” or “rapid approach*” or “rapid synthesis” OR “rapid syntheses” OR “rapid evidence assess*” OR “evidence summar*” ) OR AB ( “realist review*” OR “realist synthesis” OR “realist syntheses” OR “realist evaluation” OR “meta-method*” OR “meta method*” OR “realist approach*” OR “meta-evaluation*” OR “meta evaluation*” OR “rapid literature review*” OR “rapid systematic review*” or “rapid scoping review*” OR “rapid review*” or “rapid approach*” or “rapid synthesis” OR “rapid syntheses” OR “rapid evidence assess*” OR “evidence summar*” )  **Limiters** - Published Date: 20040101-20151231 |
| **EMBASE (Ovid)**   1. (realist review$ or realist synthesis or realist syntheses or realist evaluation or meta-method$ or meta method$ or realist approach$ or meta-evaluation$ or meta evaluation$ or rapid literature review$ or rapid systematic review$ or rapid scoping review$ or rapid review$ or rapid approach$ or rapid synthesis or rapid syntheses or rapid evidence assess$ or evidence summar$).mp. [mp=title, abstract, subject headings, heading word, drug trade name, original title, device manufacturer, drug manufacturer, device trade name, keyword] 2. limit 1 to yr="2004 -Current" |
| **Health Systems Evidence**^[[1]](#footnote-1)^  (realist review OR realist synthesis OR realist syntheses OR realist evaluation OR meta-method OR meta method OR realist approach OR meta-evaluation OR meta evaluation OR rapid literature review OR rapid systematic review OR rapid scoping review OR rapid review OR rapid approach OR rapid synthesis OR rapid syntheses OR rapid evidence assess OR evidence summary) In title or abstract. |
| **LILACS (BVSalud)**  (tw:(realist review*)) OR (tw:(realist synthesis)) OR (tw:(realist syntheses)) OR (tw:(realist evaluation)) OR (tw:(meta-method*)) OR (tw:(meta method*)) OR (tw:(realist approach*)) OR (tw:(meta-evaluation*)) OR (tw:(meta evaluation*)) OR (tw:(rapid literature review*)) OR (tw:(rapid systematic review*)) OR (tw:(rapid scoping review*)) OR (tw:(rapid review*)) OR (tw:(rapid approach*)) OR (tw:(rapid synthesis)) OR (tw:(rapid syntheses)) OR (tw:(rapid evidence assess*)) OR (tw:(evidence summar*)) AND (instance:"regional") AND ( db:("LILACS") AND year_cluster:("2011" OR "2010" OR "2013" OR "2009" OR "2012" OR "2008" OR "2007" OR "2014" OR "2006" OR "2005" OR "2004")) |
| **MEDLINE (Ovid)**   1. (realist review$ or realist synthesis or realist syntheses or realist evaluation or meta-method$ or meta method$ or realist approach$ or meta-evaluation$ or meta evaluation$ or rapid literature review$ or rapid systematic review$ or rapid scoping review$ or rapid review$ or rapid approach$ or rapid synthesis or rapid syntheses or rapid evidence assess$ or evidence summar$).mp. [mp=title, abstract, subject headings, heading word, drug trade name, original title, device manufacturer, drug manufacturer, device trade name, keyword] 2. limit 1 to yr="2004 -Current" |
| Website and search terms |
| **Google**  Rapid systematic review OR rapid review OR realist review OR rapid synthesis OR rapid evidence |
| **Google Scholar**  Rapid systematic review OR rapid review OR realist review OR rapid synthesis OR rapid evidence |

1. The search of Health Systems Evidence was conducted by Kaelan Moat, McMaster Health Forum, McMaster University, to enable all relevant records to be viewed and imported into the EndNote database. [↑](#footnote-ref-1)
