# Supplementary material for: What are the best methodologies for rapid reviews of the research evidence for evidence-informed decision making in health policy and practice: a rapid review
Source: Health Res Policy Syst. 2016 Nov 25;14:83. doi: 10.1186/s12961-016-0155-7 (PMC5123411; doi:10.1186/s12961-016-0155-7)
Supplement: Additional file 3: — Characteristics of excluded studies and reference details. (DOCX 40 kb) [file 12961_2016_155_MOESM3_ESM.docx]

## Additional file 3. Characteristics of excluded studies and reference details

### Table 1. Excluded studies (n=75)

| Study | Reason for Exclusion |
| --- | --- |
| Anon. [2014](#_ENREF_1) | Intervention - not rapid evidence synthesis |
| [Bambra et al. 2010](#_ENREF_2) | Outcomes – not relevant |
| [Barbui et al. 2010](#_ENREF_3) | Study type - not an evaluation; Intervention - not rapid evidence synthesis; Outcomes – not relevant |
| [Bearman and Dawson 2013](#_ENREF_4) | Study type - not an evaluation; Intervention - not rapid evidence synthesis |
| [Booth et al. 2013](#_ENREF_5) | Study type - not an evaluation |
| [Brener et al. 2014](#_ENREF_6) | Study type - not an evaluation |
| [Buendía-Rodríguez and Sánchez-Villamil 2006](#_ENREF_7) | Study type - not an evaluation; Intervention - not rapid evidence synthesis; Outcomes – not relevant |
| [Carrey et al. 2014](#_ENREF_8) | Intervention - not rapid evidence synthesis |
| [Chambers and Wilson 2012](#_ENREF_9) | Study type - not an evaluation; |
| [Chang et al. 2010](#_ENREF_10) | Study type - not an evaluation |
| [Cowey et al. 2010](#_ENREF_11) | Intervention - best practice statement on end of life stroke care; Outcomes – not relevant |
| [Davidson et al. 2013](#_ENREF_12) | Intervention - not rapid evidence synthesis |
| [Davino-Ramaya et al. 2012](#_ENREF_13) | Study type - not an evaluation |
| [Dixon-Woods et al. 2005](#_ENREF_14) | Study type - not an evaluation; Intervention - not rapid evidence synthesis |
| [Ellen et al. 2014](#_ENREF_15) | Intervention - summaries of systematic reviews, not rapid evidence synthesis |
| [Esandi et al. 2010](#_ENREF_16) | Intervention - not rapid evidence synthesis |
| [Franek 2013](#_ENREF_17) | Study type - not an evaluation |
| [Gough 2013](#_ENREF_18) | Study type - not an evaluation |
| [Grant and Booth 2009](#_ENREF_19) | Study type - narrative review, not an evaluation |
| [Gush and Borriello 2007](#_ENREF_20) | Study type - commentary, not an evaluation; Intervention - description of a rapid review panel |
| [Harker and Kleijnen 2011](#_ENREF_21) | Abstract of an included systematic review (Harker et al 2012). |
| [Hartz 2012](#_ENREF_23) | Study type - opinion piece, not an evaluation. |
| [Hewitt et al. 2012](#_ENREF_24) | Study type - not an evaluation; Intervention - not rapid evidence synthesis |
| [Iams 2004](#_ENREF_25) | Study type - not an evaluation; Intervention - not rapid evidence synthesis |
| [Ireland 2013](#_ENREF_26) | Study type - not an evaluation |
| [Kaltenthaler et al. 2012](#_ENREF_27) | Intervention - not rapid evidence synthesis |
| [Kamerling 2012](#_ENREF_28) | Study type - not an evaluation; Intervention - not rapid evidence synthesis |
| [Kastner et al. 2012](#_ENREF_29) | Study type - protocol for a systematic review |
| [Khan and Coomarasamy 2004](#_ENREF_30) | Study type - not an evaluation. |
| [Khangura et al. 2012](#_ENREF_31) | Study type - not an evaluation |
| [Koster et al. 2013](#_ENREF_32) | Study type - conference discussion topic. |
| [Lee et al. 2012](#_ENREF_33) | Intervention - not rapid evidence synthesis |
| [Leonard et al. 2011](#_ENREF_34) | Intervention - not rapid evidence synthesis |
| [Mahon et al. 2013](#_ENREF_35) | Intervention - not rapid evidence synthesis; Outcomes – not relevant |
| [Mays et al. 2005](#_ENREF_36) | Study type - not an evaluation; Intervention - not rapid review |
| [McCormack et al. 2007a](#_ENREF_37) | Intervention - not rapid evidence synthesis |
| [McCormack et al. 2007b](#_ENREF_38) | As above (same study) |
| [McCormack et al. 2007c](#_ENREF_39) | As above (same study) |
| [McCormack et al. 2007d](#_ENREF_40) | As above (same study) |
| [McGee and Clark 2010](#_ENREF_41) | Study type - not an evaluation |
| [McGowan et al. 2010](#_ENREF_43) | Intervention - not rapid evidence synthesis, summary of one systematic review. |
| [Merlin et al. 2014](#_ENREF_44) | Study type - mapping exercise, not an evaluation. |
| [Moher et al. 2000](#_ENREF_45) | Year of study; Intervention - not rapid evidence synthesis but testing of a method that could contribute. |
| [Muhlhauser et al. 2011](#_ENREF_46) | Language - German; Study type - not an evaluation; Intervention - not rapid evidence synthesis. |
| [Pawson et al. 2014](#_ENREF_47) | Study type - narrative review, not an evaluation; Intervention - not rapid evidence synthesis |
| [Pawson et al. 2005](#_ENREF_48) | Intervention - not rapid evidence synthesis |
| [Pawson et al. 2010](#_ENREF_49) | Study type - not an evaluation; Intervention - not rapid evidence synthesis |
| [Pearson and Coomber 2010](#_ENREF_50) | Intervention - not rapid evidence synthesis |
| [Petticrew et al. 2013](#_ENREF_51) | Study type - not an evaluation; Intervention - not rapid evidence synthesis |
| [Revere et al. 2004](#_ENREF_52) | Study type - not an evaluation; Intervention - not rapid evidence synthesis |
| [Riley et al. 2012](#_ENREF_53) | Intervention - not rapid evidence synthesis; Outcomes – not relevant |
| [Robeson et al. 2010](#_ENREF_54) | Study type - not an evaluation; Intervention - not rapid evidence synthesis |
| [Rosenbaum et al. 2008](#_ENREF_55) | Intervention - summary of a single systematic review, not rapid evidence synthesis |
| [Rosenbaum et al. 2011](#_ENREF_56) | Intervention - summary of a single systematic review, not rapid evidence synthesis |
| [Rotstein and Laupacis 2004](#_ENREF_57) | Intervention - not rapid evidence synthesis; Outcomes – not relevant |
| [Rovers and van der Heijden 2010](#_ENREF_58) | Study type - not an evaluation; Intervention - not rapid evidence synthesis |
| [Rycroft-Malone et al. 2012](#_ENREF_59) | Intervention - not rapid evidence synthesis |
| [Saul et al. 2013](#_ENREF_60) | Study type - not an evaluation. |
| [Schunemann et al. 2007](#_ENREF_61) | Study type - not an evaluation. |
| [Sheikh et al. 2007](#_ENREF_62) | Intervention - not rapid evidence synthesis |
| [Silvestre et al. 2011](#_ENREF_63) | Study type - not an evaluation; Intervention - not rapid evidence synthesis |
| [Stansfield 2011](#_ENREF_64) | Intervention - not rapid evidence synthesis; Outcomes – not relevant. |
| [Stansfield et al. 2010](#_ENREF_65) | Intervention - not rapid evidence synthesis; Outcomes – not relevant |
| [Thigpen et al. 2012](#_ENREF_66) | Study type - not an evaluation; Intervention - not rapid evidence synthesis. |
| [Thomas et al. 2013](#_ENREF_67) | Study type - not an evaluation |
| [Trevena et al. 2007](#_ENREF_68) | Intervention - not rapid evidence synthesis |
| [Tricco et al. 2011](#_ENREF_69) | Study type - not an evaluation; Intervention - not rapid evidence synthesis |
| [Turner et al. 2013](#_ENREF_70) | Intervention - not rapid evidence synthesis |
| [Varonen et al. 2001](#_ENREF_71) | Year of study – published before 2004 |
| [Westphal et al. 2014](#_ENREF_72) | Intervention - not rapid evidence synthesis |
| [Wong et al. 2013](#_ENREF_73) | Intervention - not rapid evidence synthesis |
| [Wong et al. 2012](#_ENREF_74) | Study type - not an evaluation; Intervention - not rapid evidence synthesis |
| [Wyer and Rowe 2007](#_ENREF_75) | Study type - not an evaluation |
| [Young et al. 2014a](#_ENREF_76) | Intervention - summary of a single systematic review, not rapid evidence synthesis. |
| [Young et al. 2014b](#_ENREF_77) | Study type - not an evaluation |

### References: Excluded studies (n=75)

Anon. 2014. Does therapeutic writing help people with long term conditions? Systematic review, realist synthesis and economic modelling (Project record). *Health Technology Assessment Database* [Online]. Available: http://onlinelibrary.wiley.com/o/cochrane/clhta/articles/HTA-32013001065/frame.html; http://www.nets.nihr.ac.uk/projects/hta/117001.

Bambra C, Joyce KE, Bellis MA, Greatley A, Greengross S, Hughes S, et al. 2010. Reducing health inequalities in priority public health conditions: using rapid review to develop proposals for evidence-based policy. *Journal of Public Health* 32; 496-505.

Barbui C, Dua T, van Ommeren M, Yasamy MT, Fleischmann A, Clark N, et al. 2010. Challenges in developing evidence-based recommendations using the grade approach: The case of mental, neurological, and substance use disorders. *PLoS Medicine* 7.

Bearman M and Dawson P 2013. Qualitative synthesis and systematic review in health professions education. *Medical Education* 47; 252-60.

Booth A, Harris J, Croot E, Springett J, Campbell F and Wilkins E 2013. Towards a methodology for cluster searching to provide conceptual and contextual "richness" for systematic reviews of complex interventions: case study (CLUSTER). *BMC Medical Research Methodology* 13; 118.

Brener SS, Nikitovic M, Chambers A, Ghazipura M, Schaink AK, Lambrinos AI, et al. 2014. Evidence-based practice recommendations: Health quality Ontario's approach. *Value in Health* 17; A29.

Buendía-Rodríguez JA and Sánchez-Villamil JP 2006. Using systematic reviews for evidence-based health promotion: basic methodology issues. *Rev Salud Publica (Bogota)* 8; 94-105.

Carrey NJ, Curran JA, Greene R, Nolan A and McLuckie A 2014. Embedding mental health interventions in early childhood education systems for at-risk preschoolers: an evidence to policy realist review. *Systematic Reviews* 3; 84.

Chambers D and Wilson P 2012. A framework for production of systematic review based briefings to support evidence-informed decision-making. *Systematic Reviews* 1; 32.

Chang LW, Kennedy CE, Kennedy GE, Lindegren ML, Marston BJ, Kaplan JE, et al. 2010. Developing WHO guidelines with pragmatic, structured, evidence-based processes: A case study. *Global Public Health* 5; 395-412.

Cowey E, Smith LN, Campbell L, Chalmers C, Dennis M, Fraser H, et al. 2010. Rapid Evidence Assessment (REA) as a method for developing a best practice statement on end of life care in acute stroke. *International Journal of Stroke* 5; 64.

Davidson EM, Liu JJ, Bhopal R, White M, Johnson MR, Netto G, et al. 2013. Behavior change interventions to improve the health of racial and ethnic minority populations: a tool kit of adaptation approaches. *Milbank Quarterly* 91; 811-51.

Davino-Ramaya C, Krause LK, Robbins CW, Harris JS, Koster M, Chan W, et al. 2012. Transparency matters: Kaiser Permanente's National Guideline Program methodological processes. *Permanente Journal* 16; 55-62.

Dixon-Woods M, Agarwal S, Jones D, Young B and Sutton A 2005. Synthesising qualitative and quantitative evidence: a review of possible methods. *Journal of Health Services Research & Policy* 10; 45-53.

Ellen ME, Lavis JN, Wilson MG, Grimshaw J, Haynes RB, Ouimet M, et al. 2014. Health system decision makers' feedback on summaries and tools supporting the use of systematic reviews: a qualitative study. *Evidence & Policy: A Journal of Research, Debate and Practice* 10; 337-359.

Esandi ME, De Luca M, Chapman E, García Dieguez M, Carbonelli N and Ortiz Z 2010. [Estrategias para la reducción de la brecha del conocimiento a la acción en Argentina: la prevención y tratamiento de la influenza A (H1N1) en embarazadas como caso de análisis]. Knowledge to action gap reduction strategies in Argentina: influenza A (H1N1) prevention and treatment in pregnant women as a case of analysis. *Bol. Acad. Nac. Med. B.Aires* 88; 97-107.

Franek J 2013. Challenges of developing rapid guidance for complex interventions. *BMJ Quality and Safety* 22; A33.

Gough D 2013. Meta-narrative and realist reviews: guidance, rules, publication standards and quality appraisal. *BMC Medicine* 11; 22.

Grant MJ and Booth A 2009. A typology of reviews: an analysis of 14 review types and associated methodologies. *Health Information & Libraries Journal* 26; 91-108.

Gush C and Borriello P 2007. An overview of the UK Department of Health's Rapid Review Panel. *Journal of Hospital Infection* 65 Suppl 2; 27-9.

Harker J and Kleijnen J. 2011. What is a rapid review? Oral presentation at the 19th Cochrane Colloquium; 2011 Oct 19-22; Madrid, Spain [abstract]. *Cochrane Database of Systematic Reviews, Supplement* [Online], Suppl. Available: http://onlinelibrary.wiley.com/o/cochrane/clcmr/articles/CMR-16516/frame.html.

Hartz ZMA 2012. Meta-evaluation of health management: Challenges for "new public health". *Ciencia e Saude Coletiva* 17; 832-834.

Hewitt G, Sims S and Harris R 2012. The realist approach to evaluation research: an introduction. *International Journal of Therapy & Rehabilitation* 19; 250-259.

Iams J 2004. SMFM will be first to use AJOG rapid review. *American Journal of Obstetrics and Gynecology* 191; 863.

Ireland B 2013. If rapid reviews are the answer, what is the question? *BMJ Quality and Safety* 22; A21.

Kaltenthaler EC, Dickson R, Boland A, Carroll C, Fitzgerald P, Papaioannou D, et al. 2012. A qualitative study of manufacturers' submissions to the UK NICE single technology appraisal process. *BMJ Open* 2; e000562.

Kamerling N 2012. The Greenberg Rapid Review: A Companion to the 7th Edition. *Spinal Cord* 50; 481-481.

Kastner M, Tricco AC, Soobiah C, Lillie E, Perrier L, Horsley T, et al. 2012. What is the most appropriate knowledge synthesis method to conduct a review? Protocol for a scoping review. *BMC Medical Research Methodology* 12; 114.

Khan KS and Coomarasamy A 2004. Searching for evidence to inform clinical practice. *Current Obstetrics and Gynaecology* 14; 142-146.

Khangura S, Konnyu K, Cushman R, Grimshaw J and Moher D 2012. Evidence summaries: the evolution of a rapid review approach. *Systematic Reviews* 1; 10.

Koster M, Garritty C, Gallagher C, Schunemann H and Norris S 2013. The role of rapid systematic reviews for development of rapid guidance in health care and health policy settings. *BMJ Quality and Safety* 22; A6.

Lee E, Dobbins M, Decorby K, McRae L, Tirilis D and Husson H 2012. An optimal search filter for retrieving systematic reviews and meta-analyses. *BMC Medical Research Methodology* 12; 51.

Leonard SA, Brooks-Rooney C, Kusel J and Costello S 2011. To what extent does advice from the Scottish medicines consortium (SMC) agree with that published by nice? *Value in Health* 14 (7); A355.

Mahon S, Redmond S and Carney P 2013. Transparency of the medicines reimbursement system in Ireland: A quantative analysis of the influences on reimbursement decisions [2006-2013]. *Value in Health* 16 (7); A459.

Mays N, Pope C and Popay J 2005. Systematically reviewing qualitative and quantitative evidence to inform management and policy-making in the health field. *Journal of Health Services Research and Policy* 10; 6-20.

McCormack B, Wright J, Dewar B, Harvey G and Ballantine K 2007a. A realist synthesis of evidence relating to practice development: findings from telephone interviews and synthesis of the data. *Practice Development in Health Care* 6; 56-75.

McCormack B, Wright J, Dewar B, Harvey G and Ballantine K 2007b. A realist synthesis of evidence relating to practice development: findings from the literature analysis. *Practice Development in Health Care* 6; 25-55.

McCormack B, Wright J, Dewar B, Harvey G and Ballantine K 2007c. A realist synthesis of evidence relating to practice development: methodology and methods. *Practice Development in Health Care* 6; 5-24.

McCormack B, Wright J, Dewar B, Harvey G and Ballantine K 2007d. A realist synthesis of evidence relating to practice development: recommendations. *Practice Development in Health Care* 6; 76-80.

McGee S and Clark E 2010. Creating quality evidence summaries on a clinician's schedule. *Journal of Nursing Administration* 40; 7-9.

McGowan J, Hogg W, Rader T, Salzwedel D, Worster D, Cogo E, et al. 2010. A rapid evidence-based service by librarians provided information to answer primary care clinical questions. *Health Information & Libraries Journal* 27; 11-21.

Merlin T, Tamblyn D and Ellery B 2014. What's in a name? developing definitions for common health technology assessment product types of the international network of agencies for health technology assessment (Inahta). *International Journal of Technology Assessment in Health Care* 761.

Moher D, Pham, Klassen TP, Schulz KF, Berlin JA, Jadad AR, et al. 2000. What contributions do languages other than English make on the results of meta-analyses? *Journal of Clinical Epidemiology* 53; 964-972.

Muhlhauser I, Lenz M and Meyer G 2011. Development, appraisal and synthesis of complex interventions - A methodological challenge. [German]. *Zeitschrift fur Evidenz, Fortbildung und Qualitat im Gesundheitswesen* 105; 751-761.

Pawson R, Greenhalgh J, Brennan C and Glidewell E 2014. Do reviews of healthcare interventions teach us how to improve healthcare systems? *Social Science and Medicine* 114; 129-37.

Pawson R, Greenhalgh T, Harvey G and Walshe K 2005. Realist review--a new method of systematic review designed for complex policy interventions. *Journal of Health Services Research & Policy* 10; 21-34.

Pawson R, Owen L and Wong G 2010. Legislating for health: Locating the evidence. *Journal of Public Health Policy* 31; 164-177.

Pearson M and Coomber R 2010. The challenge of external validity in policy-relevant systematic reviews: a case study from the field of substance misuse. *Addiction* 105; 136-45.

Petticrew M, Rehfuess E, Noyes J, Higgins JP, Mayhew A, Pantoja T, et al. 2013. Synthesizing evidence on complex interventions: how meta-analytical, qualitative, and mixed-method approaches can contribute. *Journal of Clinical Epidemiology* 66; 1230-43.

Revere D, Fuller S, Bugni PF and Martin GM 2004. An information extraction and representation system for rapid review of the biomedical literature. *Studies in Health Technology and Informatics* 107; 788-92.

Riley B, Norman CD and Best A 2012. Knowledge integration in public health: A rapid review using systems thinking. *Evidence and Policy* 8; 417-431.

Robeson P, Dobbins M, DeCorby K and Tirilis D 2010. Facilitating access to pre-processed research evidence in public health. *BMC Public Health* 10; 95.

Rosenbaum S, Glenton C and Oxman A. 2008. Summaries of evidence for health policymakers in low and middle income countries (LMIC). Oral presentation at the 16th Cochrane Colloquium: Evidence in the era of globalisation; 2008 Oct 3-7; Freiburg, Germany [abstract]. *Zeitschrift fur Evidenz, Fortbildung und Qualitat im Gesundheitswesen* [Online], 102. Available: http://onlinelibrary.wiley.com/o/cochrane/clcmr/articles/CMR-12368/frame.html.

Rosenbaum SE, Glenton C, Wiysonge CS, Abalos E, Mignini L, Young T, et al. 2011. Evidence summaries tailored to health policy-makers in low- and middle-income countries. *Bulletin of The World Health Organization* 89; 54-61.

Rotstein D and Laupacis A 2004. Differences between systematic reviews and health technology assessments: a trade-off between the ideals of scientific rigor and the realities of policy making. *International Journal of Technology Assessment in Health Care* 20; 177-83.

Rovers MM and van der Heijden GJ 2010. Translating research evidence into action in daily practice. *Otolaryngology - Head and Neck Surgery* 142; 29-30.

Rycroft-Malone J, McCormack B, Hutchinson AM, DeCorby K, Bucknall TK, Kent B, et al. 2012. Realist synthesis: illustrating the method for implementation research. *Implementation Science* 7; 33.

Saul JE, Willis CD, Bitz J and Best A 2013. A time-responsive tool for informing policy making: rapid realist review. *Implementation Science* 8; 103.

Schunemann HJ, Hill SR, Kakad M, Vist GE, Bellamy R, Stockman L, et al. 2007. Transparent development of the WHO rapid advice guidelines. *PLoS Medicine* 4; e119.

Sheikh L, Johnston S, Thangaratinam S, Kilby MD and Khan KS 2007. A review of the methodological features of systematic reviews in maternal medicine. *BMC Medicine* 5; 10.

Silvestre MA, Dans LF and Dans AL 2011. Trade-off between benefit and harm is crucial in health screening recommendations. Part II: evidence summaries. *Journal of Clinical Epidemiology* 64; 240-9.

Stansfield C. 2011. Locating evidence for developing countries: a case study of three public health reviews. Poster presentation at the 19th Cochrane Colloquium; 2011 Oct 19-22; Madrid, Spain [abstract]. *Cochrane Database of Systematic Reviews, Supplement* [Online], Suppl. Available: http://onlinelibrary.wiley.com/o/cochrane/clcmr/articles/CMR-16617/frame.html.

Stansfield C, Kavanagh J and Thomas J. 2010. 'Clustering' documents automatically to support scoping reviews of research. Poster presentation at the Joint Cochrane and Campbell Colloquium; 2010 Oct 18-22; Keystone, Colorado, USA [abstract]. *Cochrane Database of Systematic Reviews, Supplement* [Online], Suppl. Available: http://onlinelibrary.wiley.com/o/cochrane/clcmr/articles/CMR-15620/frame.html.

Thigpen S, Puddy RW, Singer HH and Hall DM 2012. Moving knowledge into action: developing the rapid synthesis and translation process within the interactive systems framework. *American Journal of Community Psychology* 50; 285-94.

Thomas J, Newman M and Oliver S 2013. Rapid evidence assessments of research to inform social policy: taking stock and moving forward. *Evidence & Policy: A Journal of Research, Debate and Practice* 9; 5-27.

Trevena LJ, Irwig L, Isaacs A and Barratt A 2007. GPs want tailored, user friendly evidence summaries--a cross sectional study in New South Wales. *Australian Family Physician* 36; 1065-9.

Tricco AC, Tetzlaff J and Moher D 2011. The art and science of knowledge synthesis. *Journal of Clinical Epidemiology* 64; 11-20.

Turner RM, Bird SM and Higgins JP 2013. The impact of study size on meta-analyses: examination of underpowered studies in Cochrane reviews. *PloS One* 8; e59202.

Varonen H, Kunnamo I and Stancliffe R. 2001. Grading strength of evidence from A to D for EBM guidelines statements: strength of evidence in summaries of Cochrane reviews [abstract]. *Ninth Annual Cochrane Colloquium; 2001 Oct 9-13; Lyon, France.* [Online]. Available: http://onlinelibrary.wiley.com/o/cochrane/clcmr/articles/CMR-3746/frame.html.

Westphal A, Kriston L, Holzel LP, Harter M and von Wolff A 2014. Efficiency and contribution of strategies for finding randomized controlled trials: a case study from a systematic review on therapeutic interventions of chronic depression. *Journal of Public Health Research* 3; 177.

Wong G, Greenhalgh T, Westhorp G, Buckingham J and Pawson R 2013. RAMESES publication standards: realist syntheses. *BMC Medicine* 11; 21.

Wong G, Greenhalgh T, Westhorp G and Pawson R 2012. Realist methods in medical education research: what are they and what can they contribute? *Medical Education* 46; 89-96.

Wyer PC and Rowe BH 2007. Evidence-based reviews and databases: are they worth the effort? Developing evidence summaries for emergency medicine. *Academic Emergency Medicine* 14; 960-4.

Young I, Kerr A, Waddell L, Pham MT, Greig J, McEwen SA, et al. 2014a. A guide for developing plain-language and contextual summaries of systematic reviews in agri-food public health. *Foodborne Pathogens and Disease* 11; 930-7.

Young I, Waddell L, Sanchez J, Wilhelm B, McEwen SA and Rajic A 2014b. The application of knowledge synthesis methods in agri-food public health: recent advancements, challenges and opportunities. *Preventive Veterinary Medicine* 113; 339-55.

### References to evaluation studies that did not proceed to data extraction (n=12)

Cameron A, Watt A, Lathlean T and Sturm L 2007. Rapid versus full systematic reviews: an inventory of current methods and practice in Health Technology Assessment. ASERNIP-S Report No. 60. Adelaide, South Australia: ASERNIP-S, Royal Australasian College of Surgeons.

Canadian Agency for Drugs and Technologies in Health 2010. Transcatheter aortic valve implantation: a critical appraisal of a health technology assessment and comparison with a rapid review. Ottawa: Canadian Agency for Drugs and Technologies in Health (CADTH).

Hailey D 2009. A preliminary survey on the influence of rapid health technology assessments. *International Journal of Technology Assessment in Health Care* 25; 415-418.

Jordan J, Stevenson K and Lewis R. 2011. Simple 'quick' searches to answer questions arising in clinical practice. Poster presentation at the 19th Cochrane Colloquium; 2011 Oct 19-22; Madrid, Spain [abstract]. *Cochrane Database of Systematic Reviews, Supplement* [Online], Suppl. Available: http://onlinelibrary.wiley.com/o/cochrane/clcmr/articles/CMR-16838/frame.html.

Khangura S, Polisena J, Clifford TJ, Farrah K and Kamel C 2014. Rapid review: An emerging approach to evidence synthesis in health technology assessment. *International Journal of Technology Assessment in Health Care* 30; 20-27.

Kirkland SW, Bullard M, Couperthwaite S and Rowe BH 2014. Establishing a standardized process of gathering evidence-based information to support clinical knowledge development. *Canadian Journal of Emergency Medicine* 16; S95.

Macaulay R 2014. What the english could learn from the Irish: Making the nice approval process more cost-effective. *Value in Health* 17 (7); A440.

Mijumbi RM, Oxman AD, Panisset U and Sewankambo NK 2014. Feasibility of a rapid response mechanism to meet policymakers' urgent needs for research evidence about health systems in a low income country: a case study. *Implementation Science* 9; 114.

Rizzo M, Llewellyn A and Martin A. 2011. A rapid systematic review versus a Cochrane systematic review: an empirical comparison. Poster presentation at the 19th Cochrane Colloquium; 2011 Oct 19-22; Madrid, Spain [abstract]. *Cochrane Database of Systematic Reviews, Supplement* [Online], Suppl. Available: http://onlinelibrary.wiley.com/o/cochrane/clcmr/articles/CMR-16706/frame.html.

Sagliocca L, De Masi S, Ferrigno L, Mele A and Traversa G 2013. A pragmatic strategy for the review of clinical evidence. *Journal of Evaluation in Clinical Practice* 19; 689-96.

Van de Velde S, De Buck E, Dieltjens T and Aertgeerts B 2011. Medicinal use of potato-derived products: conclusions of a rapid versus full systematic review. *Phytotherapy Research* 25; 787-8.

Warren V 2007. Health technology appraisal of interventional procedures: comparison of rapid and slow methods. *Journal of Health Services Research & Policy* 12; 142-6.

Watt A, Cameron A, Sturm L, Lathlean T, Babidge W, Blamey S, et al. 2008. Rapid versus full systematic reviews: validity in clinical practice? *ANZ Journal of Surgery* 78; 1037-40.
