# Supplementary material for: What are the best methodologies for rapid reviews of the research evidence for evidence-informed decision making in health policy and practice: a rapid review
Source: Health Res Policy Syst. 2016 Nov 25;14:83. doi: 10.1186/s12961-016-0155-7 (PMC5123411; doi:10.1186/s12961-016-0155-7)
Supplement: Additional file 4: — Characteristics of included studies. (DOCX 47 kb) [file 12961_2016_155_MOESM4_ESM.docx]

## Additional file 4. Characteristics of included studies

### Systematic reviews

Reviews are ordered chronologically, from most to least recent, and alphabetically within years.

| **Study** | **Objectives** | **Target population** | **Method/s tested** | **Outcomes reported** | **Date of last search** | **Included study designs & number** | **Country or region of studies** | **Results** |
| --- | --- | --- | --- | --- | --- | --- | --- | --- |
| [Featherstone et al. (2015)](#_ENREF_3), [Hartling et al. (2015)](#_ENREF_6) | To characterize rapid reviews (RRs) and similar products, to understand the context in  which rapid products are produced (e.g., end-users and purposes for rapid products), to understand methodological guidance and strategies used to make products rapid and describe how these differ from systematic review (SR) procedures, and to identify empiric evidence on the impact of methodological approaches on their reliability and validity. | Healthcare decision makers | RR – not clearly defined | Type of product;  Methods used;  Comparison of RRs and SRs. | November 2013 | 53 articles:  8 background articles;  3 studies with empiric data  12 reviews of rapid review types;  30 articles on rapid review methods. | RR products produced by 15 organizations:  3 Canada  2 UK  6 USA  3 Australia  1 Italy | We categorized rapid products into four groups based on the extent of synthesis: (1) ”inventories” list what evidence is available, and other contextual information needed to make decisions, but do not synthesize the evidence or present summaries or conclusions; (2) ”rapid responses” present the end-user with an answer based on the best available evidence (usually guidelines or SRs), but do not attempt to formally synthesize the evidence into conclusions; (3) ”rapid reviews” perform a synthesis (qualitative and/or quantitative) to provide an answer about the direction of evidence and possibly the strength of evidence; (4) “automated approaches” use databases of extracted study elements and programming to generate meta-analyses in response to user-defined queries.  Methodological approaches identified for rapid products include: searching fewer databases; limited use of grey literature; restricting the types of studies included (e.g., English only, most recent 5 years); relying on existing SRs; limiting full-text review; limiting dual review for study selection and/or data extraction; limiting data extraction; limiting risk of bias assessment or grading; minimal evidence synthesis; providing nominal conclusions or recommendations; and limiting external peer review.  There is almost no empiric evidence directly comparing results of rapid products with SRs. One of the included studies found no instances in which the essential conclusions of the rapid and the full reviews were opposed ([Cameron et al. 2007](#_ENREF_2)). |
| [Harker and Kleijnen (2012)](#_ENREF_5) | To answer the research question ‘What is a rapid review and is methodology consistent in rapid reviews of Health Technology Assessments?’ The specific objective was to systematically investigate methodology by  appraising in detail the methods utilized in the RRs for consistency, and in comparison with already established processes used in SRs such as searching, inclusion screening, QA, data extraction and data synthesis, and to investigate whether any differences in methodologies were related to estimated length of time taken to carry out RRs. | Those making HTA assessments in healthcare | RRs of HTAs | Methods used;  Time to complete. | April 2011 | 46 full RRs;  3 summaries of RRs | 27 UK  9 Belgium  7 Australia  6 Canada | There was a wide diversity of methodology, with some reviews utilizing well-established systematic review methods, but many others diversifying in one or more areas, that is searching, inclusion screening, quality assessment, data extraction, synthesis methods, report structure and number of reviewers. There was a significant positive correlation between the number of recommended review methodologies utilized and length of time taken in months. Time taken for the majority of reviews was 7-12 months, mean of all reviews 10.42 (SD 7.1) months. In total, 47% (n = 23) of the RRs did not have any clear research or clinical questions. Thirteen per cent (n = 3) had no clear aim or objective. Only one explicitly stated using the Patients, Interventions, Comparators, Outcomes (PICO) criteria to formulate their research questions. The majority (61%, n = 30) of the RRs reported that two reviewers were used to carry out the various processes while also reporting checking of data extraction by at least one other researcher, although the methodology was not always consistent. In total, 47% (n = 23) of the RRs clearly reported that they carried out a QA, including the specific methodology (i.e. checklist/source) used. |
| [Abrami et al. (2010)](#_ENREF_1) | To explore brief review practices in common use.  Which stages and components of brief reviews were reduced in scope or rigor and to what extent? | Policy makers and practitioners | RRs – defined as a review completed in a timely fashion (i.e. within six months) or defined by the authors as such. | Methods used; | Not reported | 42 RRs | Not reported | Out of 42 documents reviewed, 18 (about 43%) were not codable, primarily because no comprehensive description of the review methodology was reported. Even when a study generally fits the operational definition of a brief review, it often does not provide details about its methodology. The actual timeframe (how long the review took) was reported for 12 studies and ranged from several days to one year. We found that the search stage was least affected by the review limitations as searches tended to be well documented, although their comprehensiveness was unclear. For example, only 58% consulted the grey literature. See Table 1 for full results. |
| [Ganann et al. (2010)](#_ENREF_4) | 1. What are the methods used for rapid review?  2. Are there any comparisons of rapid versus traditional review methods for the same topic?  3. What are the implications of taking methodological shortcuts from a traditional Cochrane review? What biases increase? | Health system planners and policymakers | RRs – undefined | Nomenclature;  Methods used;  Comparison of RRs and SRs;  Implications of methods used. | October 2009* | 25 RRs;  45 methods articles | Not reported | Rapid reviews varied from three weeks to six months; various methods for speeding up the process were employed. Some limited searching by years, databases, language, and sources beyond electronic searches. Several employed one reviewer for title and abstract reviewing, full text review, methodological quality assessment, and/or data extraction phases. Within rapid review studies, accelerating the data extraction process may lead to missing some relevant information. Biases may be introduced due to shortened timeframes for literature searching, article retrieval, and appraisal. Various methodological articles were reviewed regarding the implications of the methods employed but without clear results of their impact. A review comparing rapid versus full systematic reviews found that overall conclusions did not vary greatly in cases where both rapid and full systematic reviews were conducted ([Cameron et al. 2007](#_ENREF_2)). Systematic reviews were also more likely to provide greater depth of information and detail in recommendations ([Cameron et al. 2007](#_ENREF_2)). |
| [Cameron et al. (2007)](#_ENREF_2), [Watt et al. (2008)](#_ENREF_8) | To examine the current evidence base pertaining to the methodology of rapid reviews, ^†^ including:   - identifying if there is a consistent methodology applied to the preparation   of rapid reviews   - highlighting the current state of discourse pertaining to rapid review methodology. | HTA agencies and users | RR, defined as a HTA report or SR that has taken between 1 and 6 months to produce which contains the elements of a comprehensive literature search.” | RR initiation and rationale;  Methods used; Content;  Time to complete;  Dissemination and impact;  Peer review procedures;  Quality evaluation of the RR; | March 2007 | 12 studies:  1 guideline (abstract);  3 program evaluations;  2 comparative study;  2 methods studies;  3 commentaries;  1 survey. | Not reported | None of the included studies detailed guidelines for the methodology underpinning RRs; rather, many offered examples and discussion surrounding the complexity of the area. Authors suggested restricted research questions and truncated search strategies as potential methods by which to limit the time taken to complete a review. One methods study found that search strategies that are restricted to the English language literature will often produce results that are close to those obtained from reviews based on more comprehensive searches that are free from language restrictions. The other methods study found that the Cochrane Controlled Trials Register (CCTR) is the single best source of randomized controlled trial references, with additional database searching retrieving only a small percentage of extra trials. Only one study could be identified that evaluated the validity and reliability of RRs compared to more extensive follow-up reports. In five of six cases, the conclusion reached by the RR product (‘Technotes’) was confirmed by later peer-reviewed reports. |

HTA – Health Technology Assessment; QA – quality assessment; RR – rapid review; SD – standard deviation; SR – systematic review;

* Confirmed by the lead author by email 13 May 2015.

† The report by Cameron and colleagues ([2007](#_ENREF_2)) contains 3 separate studies: 1) A survey of HTA agencies; 2) a systematic review of literature on rapid review methodology; and 3) a comparison of rapid reviews with full systematic reviews. Only the results of the systematic review (study 2) are reported here as the other two studies did not meet the inclusion criteria for this overview.

**Randomized controlled trial**

| **Study** | **Objectives** | **Target population** | **Method/s tested** | **Outcomes reported** | **Year of study** | **Study design and population size** | **Country or region of study** | **Results** |
| --- | --- | --- | --- | --- | --- | --- | --- | --- |
| [Opiyo et al. (2013)](#_ENREF_7) | To assess the effectiveness of different evidence summary formats for use in clinical guideline development. | Healthcare professionals with varied roles in neonatal and  pediatric policy and care | Three different packs were tested:   1. SR alone 2. SR with summary-of-findings tables 3. ‘Front-end’ short interpretation of the main findings and conclusions, drawn from evidence synthesis* plus a SR | 1^o^ outcome:  Proportion of correct responses  to key clinical questions;  2^o^ outcome:  Composite score - clarity of presentation and ease of locating the quality of evidence. | 2010 | 77 participants (7 did not attend the meeting and 5 did not complete the questionnaire);  Each participant was provided with evidence on all three tracer topics but randomization was used, within 5 professional strata, to ensure that all participants received one tracer-topic with packaging approach A, one with packaging approach B and one with packaging approach C. | Kenya | There were no significant differences between packs in the odds of correct responses to key clinical questions (adjusted ORs: pack B versus A 0.59, 95%CI 0.32 to 1.07; pack C versus A 0.66, 95%CI 0.36 to 1.21; table 3).  ‘Graded-entry’ formats (pack C) were associated with a higher mean composite score for clarity and accessibility of information about the quality of evidence for critical neonatal outcomes compared to systematic reviews alone (pack A) (adjusted mean difference 0.52, 95% CI 0.06 to 0.99). There was no difference in the mean composite score between SR with summary-of-finding tables (pack B) and SR alone (pack A). Findings from interviews with 16 panelists indicated that short narrative evidence reports (pack C) were preferred for the improved clarity of information presentation and ease of use.  “Our findings suggest that ‘graded-entry’ evidence summary formats may improve clarity and accessibility of research evidence in clinical guideline development.” |

CI – confidence interval; SR – systematic review;

* The evidence synthesis is described as “a locally prepared, short, contextually framed, narrative report in which the results of the systematic review (and other evidence where relevant) were described and locally relevant factors that could influence the implementation of evidence-based guideline recommendations (e.g. resource capacity) were highlighted.” The example provided (and tested in the RCT) included a Cochrane SR, an overview of systematic reviews and RCTs, and additional RCTs. The authors of the current overview of systematic reviews interpret pack C as being a ‘rapid review’ for the purposes of this overview.
