# Supplementary material for: What are the best methodologies for rapid reviews of the research evidence for evidence-informed decision making in health policy and practice: a rapid review
Source: Health Res Policy Syst. 2016 Nov 25;14:83. doi: 10.1186/s12961-016-0155-7 (PMC5123411; doi:10.1186/s12961-016-0155-7)
Supplement: Additional file 5: — Quality assessment of included studies. (DOCX 29 kb) [file 12961_2016_155_MOESM5_ESM.docx]

## Additional file 5. Quality assessment

### Table 1. Risk of bias assessment – The Cochrane Collaboration’s tool for assessing risk of bias. Study: Opiyo et al. (2013)

| **Bias** | **Review authors’ judgement** | **Support for judgement** |
| --- | --- | --- |
| ***Selection bias*** |  |  |
| Random sequence generation | Low risk | “One investigator (NO) generated the random allocation sequence (using a computer random number generator) and assigned participants to the different trial groups in sequential order.” Web appendix 2. |
| Allocation concealment | Unclear risk | All participants received each pack – recruitment for reasons other than intervention, but no methods of concealment documented in the paper. |
| ***Performance bias*** |  |  |
| Blinding of participants and personnel | Low risk | Blinding not mentioned in the paper but is not likely to have influenced the outcome. All participants received each of the 3 interventions (summary formats) but on different topics. |
| ***Detection bias*** |  |  |
| Blinding of outcome assessment | Low risk | Blinding of outcome assessment not mentioned in the paper but is not likely to have influenced the outcome. All participants received each of the 3 interventions (summary formats) but on different topics. |
| ***Attrition bias*** |  |  |
| Incomplete outcome data | Unclear risk | 65/70 (93%) participants completed questionnaires but no details provided of missing data. |
| ***Reporting bias.*** |  |  |
| Selective reporting | Low risk | The study protocol is not available. However, planned analyses and outcomes of interest mentioned in methods section of paper reported on in results. |
| ***Other bias.*** |  |  |
| Other sources of bias | Low risk | Low numbers of participants for subgroup analyses |

**Overall assessment:** Low risk of bias

### Table 2. Assessment of the included systematic reviews against the AMSTAR criteria – Methods for rapid reviews

| **No.** | **AMSTAR QUESTIONS** | **Featherstone et al. (2015), Hartling et al. (2015)** | **Harker and Kleijnen (2012)** | **Abrami et al. (2010)** | **Ganann et al. (2010)** | **Cameron et al. (2007), Watt et al. (2008)** |
| --- | --- | --- | --- | --- | --- | --- |
| 1 | Was an ‘a priori’ design provided? | Can't answer | Can't answer | Can't answer | Can't answer | Yes |
| 2 | Was there duplicate study selection and data extraction? | No | No | Can't answer | Can't answer | Can't answer |
| 3 | Was a comprehensive literature search performed? | Yes | No | Yes | Yes | Yes |
| 4 | Was the status of publication (i.e. grey literature) used as an inclusion criterion? | Yes | Yes | Yes | Yes | Yes |
| 5 | Was a list of studies (included and excluded) provided? | No | No | No | No | Yes |
| 6 | Were the characteristics of the included studies provided? | No | Yes | No | No | No |
| 7 | Was the scientific quality of the included studies assessed and documented? | No | No | No | No | No |
| 8 | Was the scientific quality of the included studies used appropriately in formulating conclusions? | Not applicable | Not applicable | Not applicable | Not applicable | Not applicable |
| 9 | Were the methods used to combine the findings of studies appropriate? | Not applicable | Not applicable | Not applicable | Not applicable | Not applicable |
| 10 | Was the likelihood of publication bias assessed? | No | No | No | No | No |
| 11 | Was the conflict of interest stated? | No | No | No | No | No |
|  | **Total number of 'yes' scores** | **2** | **2** | **2** | **2** | **4** |
