# Supplementary material for: What are the best methodologies for rapid reviews of the research evidence for evidence-informed decision making in health policy and practice: a rapid review
Source: Health Res Policy Syst. 2016 Nov 25;14:83. doi: 10.1186/s12961-016-0155-7 (PMC5123411; doi:10.1186/s12961-016-0155-7)
Supplement: Additional file 6: — Shortcuts and quality assessment of this review. (DOCX 30 kb) [file 12961_2016_155_MOESM6_ESM.docx]

## Additional file 6. Shortcuts taken in this review to make it rapid and quality assessment

## Shortcuts taken

- One reviewer screened titles and abstracts
- One reviewer extracted data with checking by a second reviewer
- Data extraction limited to key characteristics and results
- Limit placed on language of publication – English, French, Portuguese and Spanish
- Narrow time frame – studies published from 2004
- Narrative synthesis only, although meta-analysis was not possible due to heterogeneity of included studies
- Publication bias not assessed, although no clear methods available for assessing publication bias qualitatively
- External peer review of report to funder not obtained

### Assessment of this rapid review against the AMSTAR criteria

| **No.** | **AMSTAR questions** | **Assessment** |
| --- | --- | --- |
| 1 | Was an ‘a priori’ design provided? | Yes |
| 2 | Was there duplicate study selection and data extraction? | Yes |
| 3 | Was a comprehensive literature search performed? | Yes |
| 4 | Was the status of publication (i.e. grey literature) used as an inclusion criterion? | Yes |
| 5 | Was a list of studies (included and excluded) provided? | Yes |
| 6 | Were the characteristics of the included studies provided? | Yes |
| 7 | Was the scientific quality of the included studies assessed and documented? | Yes |
| 8 | Was the scientific quality of the included studies used appropriately in formulating conclusions? | Yes |
| 9 | Were the methods used to combine the findings of studies appropriate? | Yes |
| 10 | Was the likelihood of publication bias assessed? | No |
| 11 | Was the conflict of interest stated? | Yes |
|  | **Total number of 'yes' scores** | **10** |
